# Design of Single‐Molecule Multiferroics for Efficient Ultrahigh‐Density Nonvolatile Memories

**DOI:** 10.1002/advs.201801572

**Published:** 2018-11-08

**Authors:** Qing Yang, Tingting Zhong, Zhengyuan Tu, Lin Zhu, Menghao Wu, Xiao Cheng Zeng

**Affiliations:** ^1^ School of Physics and Wuhan National High Magnetic Field Center Huazhong University of Science and Technology Wuhan Hubei 430074 China; ^2^ Department of Chemistry and Department of Physics University of Nebraska‐Lincoln Lincoln NE 68588 USA

**Keywords:** ab initio calculations, cross‐point multiferroic tunneling junction arrays, multiferroic coupling, single‐molecule ferroelectrics, ultrahigh‐density perpendicular recording

## Abstract

It is known that an isolated single‐molecule magnet tends to become super‐paramagnetic even at an ultralow temperature of a few Kelvin due to the low spin switching barrier. Herein, single‐molecule ferroelectrics/multiferroics is proposed, as the ultimate size limit of memory, such that every molecule can store 1 bit data. The primary strategy is to identify polar molecules that possess bistable states, moderate switching barriers, and polarizations fixed along the vertical direction for high‐density perpendicular recording. First‐principles computation shows that several selected magnetic metal porphyrin molecules possess buckled structures with switchable vertical polarizations that are robust at ambient conditions. When intercalated within a bilayer of 2D materials such as bilayer MoS_2_ or CrI_3_, the magnetization can alter the spin distribution or can be even switched by 180° upon ferroelectric switching, rendering efficient electric writing and magnetic reading. It is found that the upper limit of areal storage density can be enhanced by four orders of magnitude, from the previous super‐paramagnetic limit of ≈40 to ≈10^6^ GB in.^−2^, on the basis of the design of cross‐point multiferroic tunneling junction array and multiferroic hard drive.

## 1 Introduction

With the relentless minimization of integrated circuit size to nanoscale, there are two major issues for the current prevailing silicon‐based random access memories (RAMs): 1) Quantum tunneling and memory wear will be aggravated, since their “0” and “1” states are not degenerate in energy. 2) The data storage will be lost upon power outage, while the continuous supply of power is a great challenge to power dissipation at nanoscale. Both issues may be settled by the ferroelectric RAMs (FeRAMs) or magnetic RAMs (MRAMs), as both are nonvolatile with equivalent “0” and “1” states. However, super‐paramagnetic behavior can still be a problem for MRAMs at nanoscale. Recall that the magnitude of exchange coupling *J* (usually < ≈10meV) and spin anisotropy *K* (usually < ≈meV) in the Heisenberg model

(1) $$H=-J\sum_{\left\langle i,j\right\rangle }\sigma_{i}\sigma_{j}-K\sum_{j}{\left(\sigma_{jz}\right)}^{2}$$

and the energy cost for spin flipping in MRAMs, *NJ* + *K*, is usually below a few tens of meV. In most cases of MRAMs, the energy cost depends mostly on the first term *J* for the exchange interaction of adjacent spins, which will be much reduced upon prolonged spin–spin distance (e.g., in diluted magnetic semiconductors^1^) or when the number of adjacent spins *N* is reduced in low‐dimensions. On the other hand, in cases of FeRAMs, both *J* and *K* may reach a much higher value (even higher than hundreds of meV). As a result, a high Curie temperature can still be maintained when the number of adjacent dipoles is reduced. For example, ferroelectricity (FE) has been recently explored in many 2D materials and revealed to be robust at ambient conditions,^2^, ^3^, ^4^, ^5^, ^6^, ^7^, ^8^, ^9^, ^10^, ^11^, ^12^, ^13^, ^14^, ^15^ as summarized in our recent review.^16^ As a comparison, the first measured Curie temperature of 2D ferromagnetism (FM) in CrI_3_ is ≈45 K^17^ and ≈10 K in Cr_2_Ge_2_Te_6_.^18^ Additionally, data writing in MRAMs is much more energy‐consuming than in FeRAMs, since FE switching via local electric field is highly efficient for high‐density data‐storage. However, traditional reading operation in FeRAMs can be destructive. Hence, multiferroic materials with coupled magnetism and FE, even though scarcely existing in nature, are highly desirable for efficient “electric writing + magnetic reading.”^19^, ^20^

In this paper, we propose a design of 0D multiferroic materials to the ultimate size limit in which every molecule can store 1 bit data. Note that for an isolated single‐molecule magnet (*N* = 0), its magnetism with a spin switching barrier of *K* (≈meV) cannot survive even at ultralow temperature of a few Kelvin, whereas for a single‐molecule FE, *K* can still be more than hundreds of meV. For the latter case, even the adjacent dipole–dipole interaction is negligible, the sizable energy barrier would allow FE to survive at ambient conditions. Previously, a variety of molecular switches^21^ with bistable or multistable states^22^, ^23^, ^24^, ^25^, ^26^ have been explored to build up molecular electronics, such as data storage and logical circuits. However, the external stimuli involved in these molecular switches are through inelastic electron injection directly into the molecules from the tip of scanning probe microscopy at the liquid nitrogen temperature. This approach is necessary to overcome the large switching barrier (e.g., >2 eV for ClAlPc),^27^ but is not so efficient and energy‐saving for data reading/writing with RAMs.

Alternatively, we attempted to build single molecular FE/multiferroic RAMs that can work at ambient condition. In such a unique system of 1 bit per molecule without interaction with adjacent dipoles, the tough issues of domain and fatigue that hamper applications of existing FeRAMs may be resolved. The key strategy is to seek polar molecules with bistable states, as well as with modest switching barriers and fixed polarization along the vertical direction (high *K*) for high‐density perpendicular recording. As a prototype, our design is based on metal porphyrin (MP) molecules, which are widely present in many biochemical molecules, such as iron porphyrin in haemoglobin for oxygen transport in the blood, and Mg porphyrin in chlorophyll for photosynthesis. Our first‐principles calculations show that some MP molecules possess buckled structures with switchable vertical polarizations, rendering single FE/multiferroic molecule possible as high‐density RAMs. Indeed, high storage density of ≈10^6^ GB in.^−2^ may be attained with a design of cross‐point multiferroic tunneling junction array (see below). When coupled with 2D materials like bilayer MoS_2_ or CrI_3_, the magnetization spin distribution or direction can be switched upon FE switching, thereby offering efficient electric writing + magnetic reading simultaneously.

## 2 Results and Discussion

**Figure 1:**
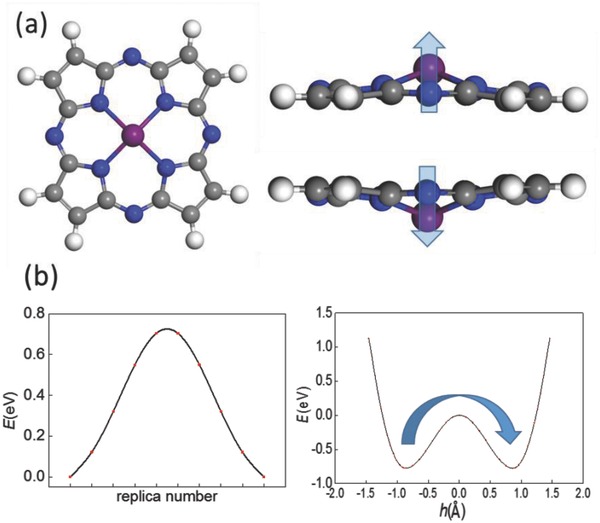
a) Geometric structures (top and side views) of buckled MP. b) Energy profile of FE switching pathway and energy dependence on *h* for ScP. Grey, blue, white, and purple spheres denote C, N, H, and M atom, respectively, and blue arrows denote the polarization directions.

Most MP molecules like FeP or MgP are strictly planar. To achieve a vertical polarization from an MP molecule, the radius of M ion should be larger than what the planar porphyrin can accommodate so that the M ion could be pushed out‐of‐plane and the molecular structure would be buckled. **Figure** **1**a displays the geometric structure of such a buckled MP molecule. For M = Sc, Ti, and V with relatively large ion radius in the 3d metal elements, their vertical distance between M ion and the porphyrin plane *h* is all greater than 0.1 Å, as listed in **Table** **1**; while for M = Cr–Zn, their planar structures are kept. Taking M = Sc as an example, the vertical distance between Sc and porphyrin plane *h* = 0.6 Å, giving rise to a vertical polarization around 0.73 e Å. The energy profile of FE switching pathway is plotted in Figure 1b, obtained based on the nudged elastic band (NEB) method, giving a moderate barrier of ≈0.73 eV. The dependence of energy on *h* also reveals an FE‐like double‐well potential. Here the FE switching barrier should be high enough for a negligible probability of quantum tunneling, or otherwise the stored data would be invalid. For a rough estimation, the transmission of a potential barrier with height *V*_0_ and width *a*: $T\;\approx \;\frac{16E(V_{0}-E)}{V_{0}{}^{2}}\;\exp\left[-\frac{2a}{\hbar }\sqrt{2m(V_{0}-E)}\right]$. Considering the case for the M ion staying at one side of the double‐well potential at 300 K, *a* = 0.8 Å, and *E* = k_B_T/2, the probability of quantum tunneling after 1 year will be only ≈10^−68^ for *V*_0_ = 0.73 eV, and ≈10^−9^ for *V*_0_ = 0.1 eV. Meanwhile a high switching barrier does not only imply a low rate of quantum‐tunneling, but also a high switching voltage and low switching speed. If we hope the applied voltage at two sides is less than 1 V, and suppose each M ion carries a charge of 2e, the desirable switching barrier should approximately range from 0.1 to 1 eV. For M = Ti and V, the barrier is only 0.04 and 0.02 eV, which may be not robust enough for practical data storage, while 4d metal M = Nb and Cd with a moderate switching barrier of, respectively, 0.33 and 0.57 eV can be good candidates. Here, each Sc or Nb ion in MP molecules possesses a magnetic moment of 1 or 3 μ_B_, which renders single‐molecule multiferroics, although the isolated magnetic moment cannot survive upon the thermal fluctuation even at a few Kelvin.

**Table 1:** Computed switching barrier Δ*E*, dipole moment *P*, and ion vertical displacement *h*, for isolated MP molecules

M	Sc	Ti	V	Cr	Nb	Cd
Δ*E* [eV]	0.73	0.04	0.02	0.0	0.33	0.57
*P* [eÅ]	0.73	0.13	0.01	0.0	0.06	0.25
*h* [Å]	0.61	0.24	0.12	0.0	0.51	0.67

**Figure 2:**
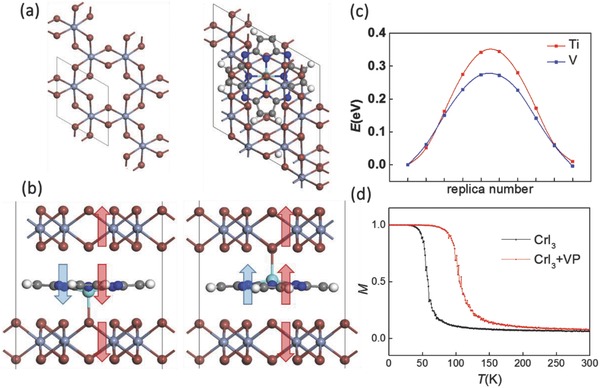
a) Overview of CrI_3_ monolayer and MP‐intercalated bilayer CrI_3_. b) The switching of net magnetization (marked by red arrows) upon FE switching (marked by blue arrows). c) Energy profile of FE switching pathway for MP‐intercalated bilayer CrI_3_ (M = Ti and V). d) Monte Carlo simulation of temperature dependence of magnetization for pristine CrI_3_ monolayer and the layer binding with VP.

For practical applications, however, MP molecules need to be fixed on substrates. As such, the polarization direction is also fixed (high *K*). In previous reports, MP molecules were usually fixed on metal or graphite surface via interfacial van der Waals interactions. If the substrates are replaced by 2D materials with anions on the surface, such as MoS_2_ or CrI_3_, the 3d metal ion in MP may attach to the surface anions like sulfur or iodine with ionic binding interaction that is much stronger than the interfacial van der Waals interaction. Taking 2D CrI_3_ as an example, when an MP molecule is placed on the surface of a CrI_3_ monolayer, the M ion tends to bind to an adjacent iodine anion, while the bistable state for FE is still missing. If an MP molecule is intercalated between two CrI_3_ layers, the M ion will bind to either side. As a result, a double‐well potential can be created, which may give rise to a switchable vertical polarization as long as the switching barrier is within the desirable range. As revealed in the FE switching pathway plotted in **Figure** **2**b and Figure S1 (Supporting Information), the switching barriers for M = Sc, Ti, V are, respectively, 1.65, 0.35, and 0.28 eV. Here TiP and VP should be switchable while the high barrier of ScP intercalated in CrI_3_ bilayer makes the system inefficient for RAMs. For the ground state structure of ScP between bilayer, due to the relatively large ion radius, each Sc ion is inclined to binding 3 iodine anions at one side, as shown in Figure S1 (Supporting Information). While for M = Ti, V, each M ion binds to only one iodine anion in the ground state, which can explain the large difference in switching barrier between ScP and TiP/VP. For TiP and VP, compared with isolated molecules, the vertical displacement of M in the intercalated bilayer systems is both enhanced to ≈0.4 Å, as listed in **Table** **2**, giving rise to a switchable vertical polarization around 0.08 eÅ. Since CrI_3_ has been experimentally verified to be intralayer ferromagnetic and interlayer anti‐ferromagnetic,^17^ the net magnetization of the bilayer CrI_3_ should be zero. Upon the intercalation of magnetic MP molecules, however, both TiP/VP will be ferromagnetically coupled to the binding layer based on our calculation. Each TiP and VP molecule possesses a magnetic moment of 2 and 3 μ_B_, respectively, and the intercalated bilayer systems keeps the same net magnetization, which can be reversed as the MP binding to the other side upon FE switching, as displayed in Figure 2b. As such, the FE and magnetism are coupled in these multiferroic 2D systems.

**Table 2:** *P* and *h* for MP‐intercalated bilayer CrI_3_ and MoS_2_

	TiP‐intercalated bilayer CrI_3_	TiP‐intercalated bilayer MoS_2_	VP‐intercalated bilayer CrI_3_	VP‐intercalated bilayer MoS_2_
*P* [eÅ]	0.08	0.39	0.08	0.30
*h* [Å]	0.43	0.42	0.40	0.40

Here, the magnetization can be reversed by 180° via electric field, which is hitherto unreported in collinear magnetic materials even though the spin distribution in some 2D multiferroic systems (e.g., C_6_N_8_H,^28^ halogen‐intercalated phosphorene bilayer^29^) has been previously predicted to be electrically tunable. The spin anisotropies of TiP and VP are both below 0.4 meV, so an isolated TiP or VP (*J* = 0) will be paramagnetic above a few Kelvin. With binding to the CrI_3_ layer, however, the magnetic moment of MP will be strongly ferromagnetically coupled (e.g., *J* = 103 meV between VP and the attached CrI_3_ monolayer), while the rise of carrier density due to charge‐transfer of MP may also result in a much enhanced Curie temperature. Based on our Monte Carlo simulation using Heisenberg model $H=-J\sum_{\left\langle i,j\right\rangle }\sigma_{i}\sigma_{j}-K\sum_{j}{\left(\sigma_{jz}\right)}^{2}$, when *J* of pristine CrI_3_ monolayer increases from 2.9 to 5.6 meV after binding with VP molecules and *K* turns to be around 1.2 meV, using 300 steps per spin based on the Metropolis algorithm, the Curie temperature also increases from ≈50 to ≈100 K, as shown in Figure 2d. In the range from 50 to 100 K where the CrI_3_ monolayer binding with VP is ferromagnetic (27 μ_B_ per supercell) while the other layer is paramagnetic (0 μ_B_ per supercell), the magnetization moment that can be electrically reversed may be further enhanced.

**Figure 3:**
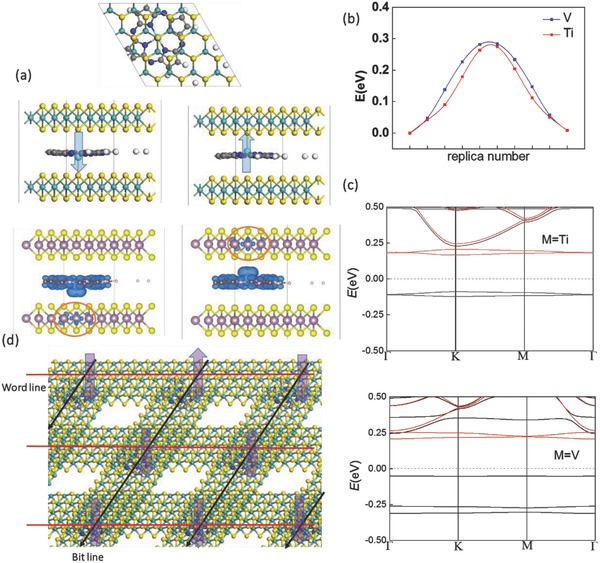
a) Geometric structure of MP intercalated bilayer MoS_2_, where the spin distribution is plotted in blue. b) FE switching pathway of TiP/VP intercalated bilayer MoS_2_. c) Band structure of TiP/VP intercalated bilayer MoS_2_. d) A design of high‐density RAMs using cross‐point‐array (CPA) structure.

The intercalation of MP molecules into bilayer MoS_2_ or other bilayer metal disulfides (e.g., WS_2_, NbSe_2_, or SnS_2_) results in a coupling of 2D semiconductor and RAMs. Note that similar coupled MP–MoS_2_ systems have already been experimentally explored as photodetectors.^30^ In any case, the intercalated MP may bind to one S atom of either layer such that a vertical polarization can be produced. Again, ScP between bilayer MoS_2_ would be inefficient for RAMs due to a large switching barrier (>1.5 eV); while for both TiP and VP, the switching barrier of MP between bilayer MoS_2_ is moderate around ≈0.3 eV (see the FE switching pathway plotted in **Figure** **3**b). The vertical polarization of TiP/VP in bilayer MoS_2_ is greatly enhanced compared with that in bilayer CrI_3_ (see Table 2), which are respectively 3.8 and 3.0 × 10^−12^ C m^−1^ in 2D, much higher than previous values predicted in bilayer BN and InSe.^31^ Meanwhile the nonmagnetic MoS_2_ layer will become magnetic and *n*‐doped with binding to the magnetic MP molecules due to charge‐transfer, as highlighted by the red circle in the spin distribution in Figure 3a. This may render an efficient approach for data reading if only the down layer is attached to the metal electrodes, similar to the previous design of 2D multiferroic transistor.^29^ The transmission will be nonmagnetic semiconducting when MP molecules bind with the upper layer, but spin‐polarized and *n*‐doped when binding with the lower layer. The band structures of TiP or VP intercalated bilayer MoS_2_ as shown in Figure 3c also turn out to be spin‐polarized with much reduced bandgap of ≈0.3 eV. For the design of high‐density RAMs using cross‐point array (CPA) structure, as shown in Figure 3d, the “read” and “write” operations are performed by commuting a cell at the crossing point between a “word” and a “bit” line. Here the word line and bit line may be composed of different metal disulfides, or the same material with different doping. Note that FE/multiferroic tunnel junctions^32^ with high on/off ratio are located between metallic electrodes with significantly different screening lengths for two distinct conductance.

**Figure 4:**
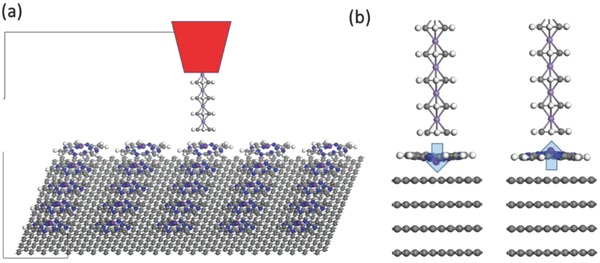
a) Design of high‐density FE hard drive. b)Model of multiferroic tunneling junction composed of ScP sandwiched between 1D Mn–benzene sandwich nanowires and 3D bulk graphite.

Apart from RAMs, the CPA structure may be unnecessary if just for high‐density data storage. In nonvolatile memories like hard drive from which high‐speed writing/reading is not required, a reading/writing head moves above the disk surface and converts the signal of each unit into a binary value. For a design of high‐density FE/multiferroic hard drive with high on/off ratio, and as a prototype of metallic electrodes with significantly different screening lengths, 1D half‐metallic Mn–benzene sandwich nanowires^33^, ^34^, ^35^, ^36^ and 3D bulk graphite are respectively selected as the top (read/write head) and bottom electrodes. As displayed in **Figure** **4**a, arrays of self‐assembly ScP molecules are adsorbed on graphite, while an Mn–benzene nanowire is used as the read/write tip. Such an FE hard disk data storage system for perpendicular recording is expected to increase the areal storage density of hard disk from the super‐paramagnetic limit of around 40 to 10^6^ GB in.^−2^. The on/off ratio can be computed by using the nonequilibrium Green's function and Landauer–Buttiker formula, implemented in the QuantumWise ATK code.^37^ When the graphite is *n*‐doped by 0.01 e per atom, for the “off” state where the polarization is downward, the transmission for spin‐up and spin‐down channel is, respectively, 3.4 × 10^−3^ and 2.6 × 10^−3^, which is nearly insulating and spin nonpolarized; for the “on” state with polarization pointing upward, as a comparison, the transmission for spin‐up and spin‐down channel is, respectively, 1.4 × 10^−1^ and 8.8 × 10^−4^, which is highly spin‐polarized (≈99.9%) and conductive. As a result, a tunneling electroresistance as high as (1.4 × 10^−1^ + 8.8 × 10^−4^)/(3.4 × 10^−3^ + 2.6 × 10^−3^) ≈2400% can be obtained, allowing efficient data reading and writing. If the graphite is *p*‐doped by 0.01 e per atom, this ratio can be further enhanced to ≈45 000%. We also note that similar polar molecular networks on graphite have already been synthesized in experiments,^27^ and the organic nanowire tip may also be substituted by tips composed of other metallic materials for more distinct screening length.

In summary, we show first‐principles evidence that certain magnetic MP molecules possess buckled structures with switchable vertical polarizations being robust at ambient conditions, rendering single‐molecule FE/multiferroics possible as efficient nonvolatile memories, while increasing the data storage density by thousands of times beyond the super‐paramagnetic limit. When intercalated in bilayer of 2D materials such as bilayer MoS_2_ or CrI_3_, the magnetization distribution or direction of MP can be switched upon FE switching, rendering efficient electric writing + magnetic reading. A high storage density of ≈10^6^ GB in.^−2^ may be obtained via a prototypical design of cross‐point multiferroic tunneling junction array and multiferroic hard drive. Similar design may be realized with other polar molecules with bistable states and with switchable dipole moments that can be fixed along the vertical direction.

## 3 Experimental Section

The theoretical calculations were performed based on density functional theory methods implemented in the Vienna ab initio Simulation Package 5.4 code.^38^, ^39^ The exchange–correlation effect was described within the generalized gradient approximation in the Perdew–Burke–Ernzerhof (PBE)^40^, ^41^ functional, together with the projector augmented wave method. The Brillouin zone integration of the supercell in 2D was sampled with a 5 × 5 × 1 Monkhorst–Pack^42^ grid, the kinetic energy cutoff was set to be 400 eV, and a vacuum space of 40 Å was set in the vertical direction. PBE‐D2 functional of Grimme^43^ was used to account for weak van der Waals interactions. The Berry‐phase method^44^ was employed to evaluate polarization, while the NEB^45^ method was used to examine the migration paths and to compute diffusion energy barrier. For intercalated bilayer system, to eliminate the interaction between adjacent molecules, a 2 × 2 supercell of CrI_3_/MoS_2_ is adopted so that the distances between the hydrogen atoms on the edges of two adjacent molecules are greater than 3.7 Å, and the interaction between them will be negligible. The binding energies of M ( = Sc, Ti, V) in MP molecules range from ≈12 to ≈14 eV per atom, much higher than the bulk cohesive energies of M, ranging from ≈4 to ≈6 eV per atom, while the binding energies of MP to bilayer MoS_2_ are all higher than 2 eV per molecule, revealing the stability of those systems.

## Conflict of Interest

The authors declare no conflict of interest.

## Supporting information

Supplementary
