# Supplementary material for: Design of Single‐Molecule Multiferroics for Efficient Ultrahigh‐Density Nonvolatile Memories
Source: Adv Sci (Weinh). 2018 Nov 8;6(1):1801572. doi: 10.1002/advs.201801572 (PMC6325569; doi:10.1002/advs.201801572)
Supplement: Supplementary file 1 — Supplementary [file ADVS-6-1801572-s001.pdf]

Copyright WILEY-VCH Verlag GmbH & Co. KGaA, 69469 Weinheim, Germany, 2018.

ADVANCED SCIENCE
Open Access

Supporting Information

for *Adv. Sci.,* DOI: 10.1002/advs.201801572

Design of Single-Molecule Multiferroics for Efficient Ultrahigh-Density Nonvolatile Memories

Qing Yang, Tingting Zhong, Zhengyuan Tu, Lin Zhu, Menghao Wu, and Xiao Cheng Zeng**

Copyright WILEY-VCH Verlag GmbH & Co. KGaA, 69469 Weinheim, Germany, 2016.

Supporting Information

Design of Single-Molecule Multiferroics for Efficient Ultrahigh-Density Non-Volatile Memories

Qing Yang, Tingting Zhong, Zhengyuan Tu, Lin Zhu, Menghao Wu*, Xiao Cheng Zeng*

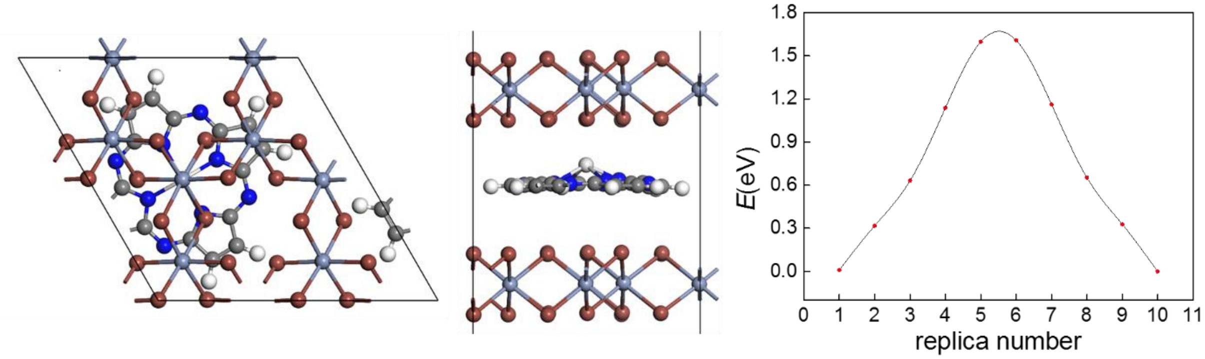

Figure S1. Geometric structures of ScP-intercalated bilayer CrI_3 and its FE switching pathway.
